# Agroinfiltration for transient gene expression and characterisation of fungal pathogen effectors in cool-season grain legume hosts

**DOI:** 10.1007/s00299-021-02671-y

**Published:** 2021-04-03

**Authors:** Johannes W. Debler, Bernadette M. Henares, Robert C. Lee

**Affiliations:** Centre for Crop and Disease Management, School of Molecular and Life Sciences, Curtin University, 1 Kent St, Bentley, WA 6102 Australia

**Keywords:** *Agrobacterium*, Phytopathogen, Plant pathogen, Necrotroph, *Dothideomycete*, *Ascochyta*

## Abstract

**Key message:**

Modified pEAQ-HT-DEST1 vectors were used for agroinfiltration in legumes. We demonstrate protein expression and export in pea, lentil, and faba bean; however, the method for chickpea was not successful.

**Abstract:**

Agroinfiltration is a valuable research method for investigating virulence and avirulence effector proteins from pathogens and pests, where heterologous effector proteins are transiently expressed in plant leaves and hypersensitive necrosis responses and other effector functions can be assessed. *Nicotiana benthamiana* is widely used for agroinfiltration and the characterisation of broad-spectrum effectors. The method has also been used in other plant species including field pea, but not yet developed for chickpea, lentil, or faba bean. Here, we have modified the pEAQ-HT-DEST1 vector for expression of 6 × histidine-tagged green-fluorescent protein (GFP) and the known necrosis-inducing broad-spectrum effector necrosis and ethylene-inducing peptide (Nep1)-like protein (NLP). Modified pEAQ-based vectors were adapted to encode signal peptide sequences for apoplast targeting of expressed proteins. We used confocal microscopy to assess the level of GFP expression in agroinfiltrated leaves. While at 3 days after infiltration in *N. benthamiana*, GFP was expressed at a relatively high level, expression in field pea and faba bean at the same time point was relatively low. In lentil, an expression level of GFP similar to field pea and faba bean at 3 days was only observed after 5 days. Chickpea leaf cells were transformed at low frequency and agroinfiltration was concluded to not be successful for chickpea. We concluded that the pEAQ vector is suitable for testing host-specific effectors in field pea, lentil, and faba bean, but low transformation efficiency limits the utility of the method for chickpea.

**Supplementary Information:**

The online version contains supplementary material available at 10.1007/s00299-021-02671-y.

## Introduction

Agroinfiltration is the process by which transgenes are transiently expressed in somatic cells of plant tissues such as leaves. *Agrobacterium tumefaciens* bacteria carrying the binary Ti-plasmid vector with a host-compatible transgene expression cassette are infiltrated into intact leaves of whole plants using a blunt-ended plastic syringe (Kapila et al. 1997; Schöb et al. 1997; Van der Hoorn et al. 2000). Cells are genetically transformed by random integration of T-DNA between left and right transformation border sequences, into the target plant nuclear DNA. Where expression of the transgene is under control of a suitable promoter, such as the Cauliflower Mosaic Virus 35S (CaMV-35S) promoter, transgene-encoded protein can be reliably produced in the agroinfiltrated plant. The native Australian solanaceous plant, *Nicotiana benthamiana*, is particularly susceptible to genetic transformation by *Agrobacterium* and is highly amenable to efficient heterologous protein synthesis using the agroinfiltration method. *N. benthamiana* plants have great potential as ‘biofactories’ for the production of proteins, with the ability to perform a range of post-translational modifications required for correct protein folding and biological activity (Bally et al. 2018). *N. benthamiana* has also provided an elegant experimental system for testing the role of effector proteins from plant pathogens with notable examples from pathogenic bacteria (Ham et al. 2008), oomycetes (Huitema et al. 2005; Mafurah et al. 2015; Chen et al. 2018; Yin et al. 2019), fungi (Van der Hoorn et al. 2000; Ma et al. 2012), nematodes (Postma et al. 2012), and insects (Rodriguez et al. 2014). Hypersensitive responses and induced necrosis in *N. benthamiana* exposed to transgene-expressed effectors suggest virulence roles of broad-spectrum effectors from the source pathogen in its natural host. Co-expression of agroinfiltrated constructs for both pathogen effector and cognate native host receptor in *N. benthamiana* has been successfully used to demonstrate effector and receptor function for proteins from pathogens outside of their natural host system. An example of this application is for the *Cladosporium fulvum* avirulence effector and tomato host–receptor pairs, *Avr*4/*Cf*-4 and *Avr*9/*Cf*-9 that when co-expressed in *Nicotiana tabacum* and *N. benthamiana* produce hypersensitive chlorosis and necrosis responses (Van der Hoorn et al. 2000). The *N. benthamiana* system has provided a robust and reliable testing platform for receptor–effector interactions for some pathosystems, and for effectors with broad host activity. However, effectors from pathogens that have narrow host specificity or may only have activity in their native host species require optimisation of agroinfiltration in the particular plant host of interest. It is important that the agroinfiltration system is tested in the many and varied plant species in which molecular pathology research is undertaken, so that we can have confidence in this experimental approach for testing candidate effector genes, and characterising validated effectors in their respective host. In addition to *N. benthamiana*, agroinfiltration has been used for *Arabidopsis thaliana* (Van der Hoorn et al. 2000; Ham et al. 2008), tomato (Postma et al. 2012; Zhang et al. 2013; Mafurah et al. 2015), lettuce (Wroblewski et al. 2005), capsicum (Chen et al. 2018) and potato (van Poppel et al. 2008). Legume species including common bean (*Phaseolus vulgaris*; Kapila et al., 1997; Suzaki et al., 2019), soybean (*Glycine max*; King et al. 2015; Suzaki et al. 2019), and pea (*Pisum sativum*; Guy et al., 2016; Klosterman et al., 2003; Van der Hoorn et al., 2000) have also been successfully agroinfiltrated. Transient β-glucuronidase (GUS) expression by agroinfiltration was demonstrated, and a pea aphid salivary protein and suspected insect effector, Ap25, was transiently expressed in pea using agroinfiltration with the effect of increasing the reproduction rate of the insect pest (Guy et al. 2016). Knowledge of pathogen effectors and their host or cultivar-specific interaction with crop plants has contributed to the concept of effector-guided resistance breeding (Vleeshouwers and Oliver 2014). Characterised pathogen effectors from oomycete and fungal diseases of potato and wheat have been used to dissect the genetic resistance mechanisms in their respective hosts and to screen breeding material in plant breeding programs (Vleeshouwers and Oliver 2014).

Our research interest is the ascochyta blight diseases of the cool-season food legume species, including field pea, chickpea (*Cicer arietinum*), lentil (*Lens culinaris*), and faba bean (*Vicia faba*). *Peyronellaea pinodes* and *Phoma koolunga* cause pea blackspot in field pea, and *Ascochyta rabiei*, *A. lentis*, and *A. fabae* cause ascochyta blights of chickpea, lentil, and faba bean, respectively (Tivoli and Banniza 2007; Davidson et al. 2009). The agroinfiltration process provides an efficient means for screening candidate genes from fungal pathogens and integrates well with recent *Ascochyta* genome sequencing projects in which candidate effector genes have been identified (Lee et al. 2021; Shah et al. 2020). In the current work, we have developed modified constructs for expression of proteins in grain legume species, based on the agroinfiltration vector pEAQ-HT-DEST1 (Sainsbury et al. 2009; Peyret and Lomonossoff 2013). The paper focuses on the demonstration of somatic cell transformation using green-fluorescent protein (GFP) expression and confocal microscopy, and the use of signal peptide sequences from legume species for the apoplast targeting of expressed proteins to validate necrosis-inducing effectors from host-specific pathogenic fungi. Delivery of plant-produced recombinant proteins to the apoplast in the experimental system that we have developed is essential to testing the role of pathogen effector protein candidates at their most likely site of action; at the apoplast side of target cell plasma membranes. In the present study, the necrosis and ethylene-inducing peptide (Nep1)-like protein (NLP) from the ascochyta-causing pathogens *P. pinodes* and *A. rabiei*, was used as a positive control for necrosis. NLP is an extracellular protein first identified from the culture filtrate of *Fusarium oxysporum* that induced ethylene production and necrosis in coca leaves (*Erythroxylum coca*) (Bailey 1995). This protein is present in various microorganisms including fungi, bacteria, and oomycetes, and causes necrosis only in dicotyledonous plants, while monocots are unaffected (Pemberton and Salmond 2004; Oome and Van den Ackerveken 2014). The mechanism by which NLP induces necrosis in dicots is yet to be determined, but based on crystal structure and mutagenesis studies of *Pythium aphanidermatum Pya*NLP, it has been proposed that NLP can act as a cytolytic toxin that induces plasma membrane leakage (Ottmann et al. 2009). Several studies have indicated that NLPs have undergone functional diversification and function as non-cytotoxic proteins that induce the host immune response (Böhm et al. 2014; Oome et al. 2014). Qutob et al. (2006) report that NLP induces cell death in dicotyledonous plants only when the protein is targeted to the apoplast side of the plasma membrane. This makes NLP a good control to test whether pathogen effector proteins and effector candidates can be delivered to the apoplast from transformed host cells to generate their necrosis-inducing effects.

## Materials and methods

### Modified pEAQ-HT-DEST1 constructs

**Fig. 1:**
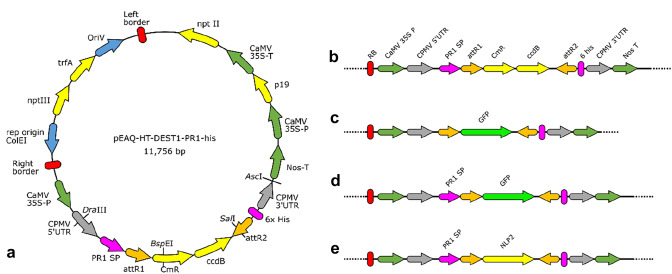
Agroinfiltration constructs (not to scale) based on the pEAQ-HT-DEST1 vector. **a**, **b** pEAQ-HT-DEST1-*Mt*PR1-His, **c** pEAQ-HT-DEST1-GFP-His (cytoplasm-localised GFP), **d** pEAQ-HT-DEST1-*Mt*PR1-GFP-His (apoplast-targeted GFP), and **e** pEAQ-HT-DEST1-*Mt*PR1-NLP2-His (apoplast-targeted NLP2)

For the construction of vectors for *Agrobacterium*-mediated transient expression in legume plants, we used the Gateway-compatible binary vector pEAQ-HT-DEST1 (GenBank: GQ497235.1) as a starting point for building constructs for production of cytoplasm-localised and apoplast-targeted proteins. We produced the modified pEAQ-HT-DEST1-based vectors, pEAQ-HT-DEST1-His, which carries nucleotide sequence encoding a C-terminal 6 × His tag, and pEAQ-HT-DEST1-PR1-His with a C-terminal His tag sequence and nucleotide sequence encoding an N-terminal signal peptide corresponding to PR-1 protein signal sequences from legumes (Fig. 1a). The PR-1 signal peptide sequences for *Medicago truncatula* and lentil were found by BLAST searches using NCBI BLAST of GenBank database for *M. truncatula* (CAA56174.1) and the Cool Season Food Legume genome database for lentil, with the *Nicotiana tabacum* PR-1a sequence as described previously for agroinfiltration in *Nicotiana spp.* (Van der Hoorn et al. 2000).

To construct pEAQ-HT-DEST1-His, the pEAQ-HT-DEST1 backbone vector was digested with *Sal*I and *Asc*I near the attR2 site and the NosT sequences, respectively, and ligation of the fragment containing the 6 × His sequence. The 6 × His fragment was synthesised and assembled by PCR using pEAQ-HT-DEST1 DNA as a template and a three-step PCR sequence. Step 1 was to amplify a fragment from the vector *Sal*I site to the attR2 sequence, with the 6 × His sequence extension encoded in the primer (primers JD14 and JD16; Supplementary Table, Table_S1). In step 2, a fragment from the vector *Asc*I site in the NosT sequence to the CPMV-HT 3′ UTR sequence with a complementary 6 × His sequence extension was produced by PCR. Fragments from steps 1 and 2 were joined together in a third PCR reaction in which annealing of the complementary His sequences and priming at the terminal *Asc*I and *Sal*I fragment ends produced a single fragment for ligation into the *Asc*I / *Sal*I-cut vector backbone (Fig. 1a). All PCR reactions were carried out in 10 µL reactions containing 0.5 µL Q5 and Phusion proof-reading *Taq* polymerase (New England Biolabs, Ipswich, MA, USA), 1 × concentration of respective PCR buffer and 0.5 µM of each primer. Amplification/cycling conditions were: denaturation for 1 min at 98 °C, 35 cycles of 10 s at 98 °C, 10 s at 55 °C and 2 min at 72 °C, final elongation for 5 min at 72 °C. Primers used are listed in Table S1.

pEAQ-HT-DEST1-*Mt*PR1-His was constructed by the addition of the PR-1 signal peptide-encoding nucleotide sequence in a gBlock gene fragment synthesised by Integrated DNA Technologies (Coralville, IA, USA). The gBlock was an 852 bp fragment corresponding to the pEAQ-HT-DEST1 sequence from the *Dra*III restriction site in the CPMV 5′ UTR sequence and the *Bsp*EI site in the chloramphenicol resistance gene (CmR) (Fig. 1a). The *M. truncatula* PR-1 signal peptide sequence (*Mt*PR1-SP; 84 bp) was encoded between CPMV 5′ UTR and attR1. Subsequent to the testing of the pEAQ-HT-DEST1-*Mt*PR1-His vector with GFP, GUS and NLP inserts, we produced a pEAQ-HT-DEST1-*Lc*PR1-His construct in which the *Mt*PR1-SP nucleotide sequence was substituted with the PR-1 signal peptide sequence, *Lc*PR1-SP from lentil (*Lens culinaris*). To produce these constructs, we ligated *Dra*III/*Bsp*EI-cut gBlocks encoding the *Lc*PR1-SP nucleotide sequence to the *Dra*III/*Bsp*EI-cut pEAQ-HT-DEST1-His construct.

All primers for PCR were from Macrogen (Seoul, South Korea), Q5 and Phusion proof-reading *Taq* polymerase was from New England Biolabs (Ipswich, MA, USA), restriction enzymes and ligation reactions used T4 DNA ligase (New England Biolabs). Restriction-digested vector DNA and PCR fragments were excised and purified from 1% agarose gels (Bioline, Alexandria, NSW, Australia) using an AccuPrep gel purification kit (Bioneer, Kew East, Australia). Plasmid vectors and modified constructs were transformed in *Escherichia coli* Top10 cells (Thermo Fisher Scientific, Waltham, MA, USA) using 42 ℃ heat shock of chemically competent cells and grown in LB. For each of the modified pEAQ-based constructs, preservation of original vector sequence and the correct orientation and reading frame for introduced DNA adjacent to the attR1 and attR2 recombination sites were confirmed by Sanger sequencing (Macrogen).

### Insertion of transgenes into pEAQ-based vectors using Gateway cloning

Entry clones for Gateway cloning were produced for the *E. coli* β-glucuronidase (GUS), *Aequoria victoria* green-fluorescent protein (GFP), and the *P. pinodes* and *A. rabiei* NLP2 cDNA sequences, which encode the second of two NLP isoforms from these species. The GFP cDNA sequence was amplified from pGpdGFP plasmid DNA. pGpdGFP is a fungal transformation construct for stable expression of GFP in fungi (Sexton and Howlett 2001). GUS sequence was amplified from a pEAQ-HT-DEST1-GUS obtained from R. Burton (University of Adelaide). Fungal NLP2 cDNAs from the field pea and chickpea ascochyta blight pathogens, *P. pinodes* isolate M074 and *A. rabiei* ME14, were amplified from cDNA synthesised using total RNA isolated from pea and chickpea leaves infected with the respective pathogens. RNA was extracted using TRIzol reagent (Invitrogen, Carlsbad, CA, USA) and cDNA synthesis was performed using the iScript cDNA synthesis kit (BioRad, Hercules, CA, USA). PCR primers for amplification of GFP and NLP2 cDNAs carried the attB1 and attB2 recognition sites for Gateway BP recombination into the Gateway entry vector, pDONR/zeo (Thermo Fisher Scientific). We performed a two-step PCR protocol to add the complete attB sites to our sequences of interest. The primers for the first PCR step had 15 bp GFP or NLP2 gene-specific sequences and 12–13 bp tails partially encoding respective attB sequences. The first-round PCR product was used in the second PCR step with the universal attB primers JD017 and JD018 carrying 12 and 13 bp complimentary sequences matching the tails of the gene-specific first-round PCR primers, and additional 17 bp oligonucleotide to complete the full attB1 and attB2 sequences. The PCR products carrying the full attB1 and attB2 sites were incubated together with pDONR/zeo that carries the respective attP1 and attP2 sites for Gateway recombination, in the presence of BP clonase II enzyme mix (Thermo Fisher Scientific). The recombination reaction mix exchanges the *ccdB* suicide gene cassette from pDONR/zeo for the attB1 and attB2-flanked gene of interest (Katzen 2007).

Gateway recombination of entry clones with the *Agrobacterium* transformation constructs, pEAQ-HT-DEST1-His, pEAQ-HT-DEST1-*Mt*PR1-His or pEAQ-HT-DEST1-*Lc*PR1-His, was undertaken using the LR clonase II Gateway recombination kit (Thermo Fisher Scientific) following the manufacturer’s protocols with modifications in regard to quantities. The modified BP and LR recombination protocols are available on https://www.protocols.io (BP recombination: dx.doi.org/10.17504/protocols.io.g5rby56 and LR recombination: dx.doi.org/10.17504/protocols.io.g5sby6e). Correct insertion of cDNA transgenes in expression clones was confirmed by Sanger sequencing. Primer sequences for amplification of GUS, GFP, and NLP2 transgenes with terminal attB1 and attB2 sequences for Gateway cloning are detailed in the Supplementary Table, Table_S1.

### *A. tumefaciens* transformation

*A. tumefaciens* GV3101::pMP90 cells were transformed by addition of 0.5 µL modified pEAQ plasmid DNA at 100 ng µL^−1^ concentration and electroporated using a BioRad Gene Pulser Xcell electroporator. Electroporated cells were plated on Yeast-Malt agar containing 30 µg mL^−1^ kanamycin, 100 µg mL^−1^ rifampicin, and 25 µg mL^−1^ gentamicin, and grown at 25 ℃ for 2–3 days until colonies appeared. Transformants were selected and verified as carrying respective modified pEAQ constructs using colony PCR with primers JD029 and JD030, which bind outside the Gateway recombination sites. *A. tumefaciens* clones were maintained as glycerol stocks in 20% glycerol at − 80 ℃.

### Agroinfiltration and confocal microscopy

*Agrobacterium* transformant clones were grown in 10 mL LB containing 50 µg mL^−1^ rifampicin and 30 µg mL^−1^ kanamycin, in 50 mL plastic Falcon tubes at 28 ℃ with 280 rpm shaking for 24 h. Cultures were centrifuged for 10 min at 4000 g at 4 ℃, resuspended in 3 mL 10 mM MES, 10 mM MgCl_2_, and further diluted to OD_600_ of 0.5 (1 cm cuvette) in 10 mM MES-MgCl_2_. Acetosyringone (Sigma-Aldrich, St Louis, MO, USA) was added from a 200 mM stock solution to a final concentration of 200 µM and *Agrobacterium* suspensions were incubated at room temperature for 3 h prior to leaf infiltration. Field pea (variety ‘Kaspa’), lentil (variety ‘PBA Ace’ and others as noted), chickpea (variety ‘PBA HatTrick’ and others as noted), faba bean (variety ‘PBA Rana’), and *Nicotiana benthamiana* (variety ‘Lab’) were grown in five cm square, 10 cm deep pots in potting soil (“UWA Mix”, RichGro, Jandakot, Australia) in growth rooms at 18 – 25 ℃ under 5000 K LED light tubes (S-Tech, Canning Vale, Australia) with a 12 h on, 12 h off regime. Leaves of 2-week-old legume seedlings or 4-week-old nicotiana plants were agroinfiltrated by placing a 1.0 mL plastic syringe against the abaxial side of leaves with a gloved finger on the adaxial side for support, and applying gentle syringe pressure to infiltrate *Agrobacterium* into the leaves via the stomata. Plants were maintained for 3–5 days post-agroinfiltration and excised for microscopy and photography. For confocal microscopy, sections of agroinfiltrated leaves were excised from areas of the leaf adjacent to the site of syringe contact with the leaf sample. Excised sections were mounted on glass slides with 20% glycerol with a cover slip and placed on the inverted stage of a Nikon A1 + confocal microscope (Nikon Instruments, Tokyo, Japan). Confocal laser-scanning microscopy was performed using four lasers with excitation wavelengths of 402 nm, 489 nm, 560 nm, and 640 nm and image capture with filters for emitted light at 450 nm, 525 nm, 595 nm, and 700 nm, for differentiation of signals from chloroplasts, cell wall autofluorescence, and GFP. Laser power and PMT gain and offset settings were constant for all confocal imaging of GUS control and GFP-expressing leaves for all species. Images for *N. benthamiana* and for some GFP expression in legumes such as for faba bean are saturated for GFP imaging. This has been done to provide context for the lower expression levels in samples such as lentil and chickpea. Z-Series images were collected using NIS-Elements software (Nikon) and two-dimensional Z-projections for publication were produced using Fiji (ImageJ) software v 1.51w. For recording of images of leaf necrosis on plant leaves expressing fungal NLP2, photographs were taken over a light box emitting fluorescent white light using a tripod-mounted digital camera (Fujifilm, Tokyo, Japan). Images for each plant tissue sample and corresponding expression construct were representative of at least three replicate leaf samples.

### Estimation of protein expression efficiency in *N. benthamiana* and field pea

To approximate the relative amounts of protein transiently expressed in *N. benthamiana* and field pea, we extracted proteins from leaves of each plant species agroinfiltrated with constructs for expression of cytoplasm-localised GUS (control), and both apoplast-targeted and cytoplasm-localised GFP, after 5 days. Plant tissue (0.3 g) was ground in a mortar and pestle with 3.0 mL 50 mM sodium phosphate buffer pH 8.0 and centrifuged at 5000 g for 10 min to remove cellular debris. GFP fluorescence of leaf extracts was measured using a BioTek Synergy HTX microplate reader (Winooski, VT, USA) in fluorescence detection mode with excitation and emission settings at 485 nm and 528 nm, respectively. Plant extract protein amounts were measured using Pierce Coomassie protein reagent (Thermo Fisher Scientific). Plant extracts and GFP estimations were performed in triplicate and statistical analysis implemented One-Way ANOVA and a Pairwise *T *Test in *R*. To estimate the specific fluorescence of recombinant GFP, we partially purified recombinant GFP from the most active, pooled fractions of cytoplasm-localised GFP from nicotiana and pea, on HisPur Nickel-NTA resin (Thermo Fisher Scientific). Protein binding to the affinity media was in 50 mM Tris–HCl buffer, pH 8.2 and elution was with Tris buffer containing 250 mM imidazole. For buffer exchange of the eluted protein fractions, we used 5 mL Nanosep 10 KDa ultrafiltration devices (Pall Corporation, New York, NY, USA). Protein concentration and fluorescence of pooled crude protein fractions and the partially purified GFP were measured as described above. Purity of the eluted GFP was assessed by SDS-PAGE (Laemmli 1970). Gel imaging and band densitometry were performed using BioRad ChemiDoc transilluminator and imaging software (BioRad, Hercules, CA, USA).

## Results

### Agroinfiltration construct preparation and *A. tumefaciens* strains

The pEAQ-HT-DEST1 vector was selected for the development of constructs for agroinfiltration in cool-season food legume species, because this vector has been optimised for high levels of transient protein expression in plants. This has been achieved in the pEAQ vector series by addition of 5′ and 3′ CPMV-HT UTR sequences and a P19 plant expression cassette for co-expression of the P19 silencing suppressor and the inserted transgene (Sainsbury et al. 2009). pEAQ-HT-DEST1 and the derived constructs produced herein have T-DNA left and right borders for integration into the nuclear genome of transformed leaf tissue cells of agroinfiltrated leaves. In addition, the pEAQ vectors have Gateway recombination sites located within the CPMV UTR sequences to enable efficient insertion of user-selected transgenes into an expression cassette under the control of the CaMV-35S promoter (Sainsbury et al. 2009). Our modification of pEAQ-HT-DEST1 consisted of first adding a C-terminal 6 × His tag after the attB2 Gateway recombination site using a three-step PCR to produce the intermediate construct pEAQ-HT-DEST1-His (Fig. 1a), thereby providing the option for purification of expressed proteins by affinity chromatography or for detection by Western blot. This construct is analogous to the pEAQ-HT-DEST3 vector produced by Sainsbury et al. (2009). Agroinfiltration relies on the transformation of somatic plant cells with DNA comprised between the pEAQ binary vector left and right T-DNA borders, and production of transgene-encoded protein in those transformed cells. Testing of fungal protein effectors in most cases depends on the delivery of these proteins to the plant apoplast. We hypothesised that the addition of an N-terminal plant secretion signal sequence to transgenes would result in transiently expressed proteins being exported from transformed cells to the leaf apoplast where their native function would be detected. Nucleotide sequence encoding the known secretion signal sequence from PR-1 protein from the model legume species *M. truncatula* was added to our pEAQ-HT-DEST1-His construct, adjacent to and upstream of the attR1 Gateway recombination site (Fig. 1a). The resulting pEAQ-HT-DEST1-*Mt*PR1-His construct (Fig. 1b) was next used for Gateway cloning of cDNA sequences for the selected transgenes, for transient expression of GFP and the known necrosis-inducing NLP2 effector from fungal pathogens of certain legume species. Vector diagrams (Fig. 1) show the locations of modifications to pEAQ-HT-DEST1.

### GFP is expressed efficiently in most legume species but not chickpea

**Fig. 2:**
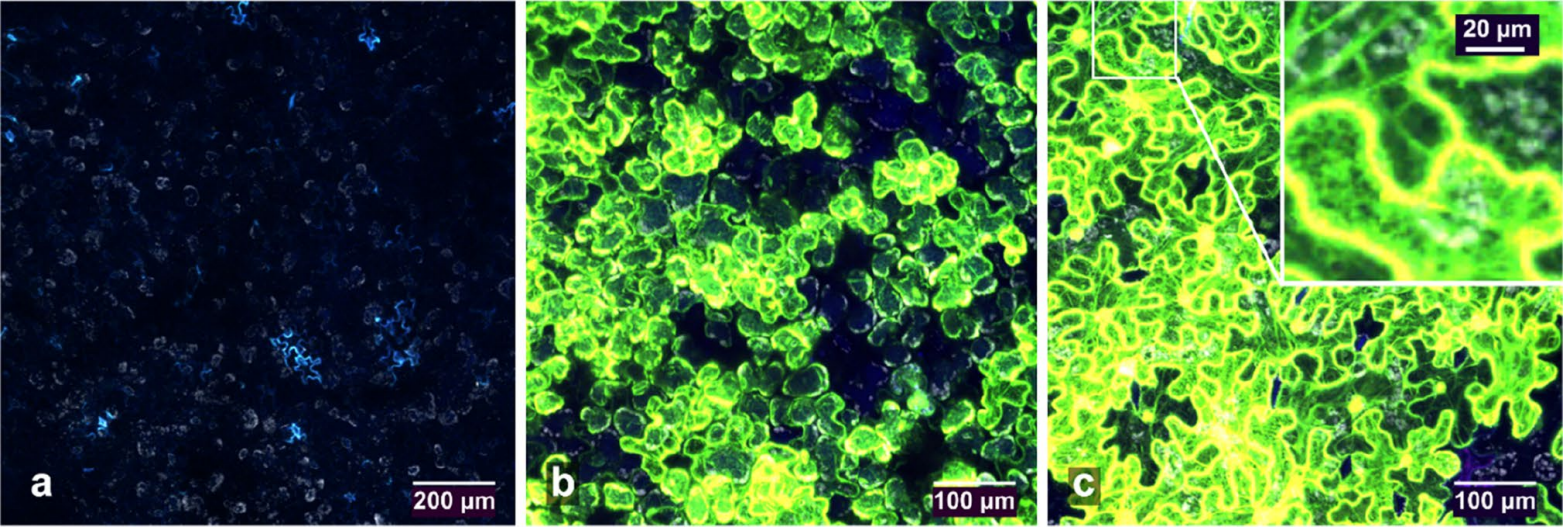
Agroinfiltration (abaxial) of *N. benthamiana* for the constitutive expression of GFP, observed using confocal microscopy at 5 days after infiltration. **a** GUS control 10 × objective. **b** Expression of GFP and localisation to cytoplasm by agroinfiltration of *A. tumefaciens* carrying GFP_cyt_ construct for non-targeted GFP. **c** Apoplast-targeted GFP expressed by agroinfiltration of *A. tumefaciens* with GFP_apo_ construct with *M. truncatula* PR-1 secretion signal sequence. **c** Inset image, enlarged section showing an apparent striated and granular appearance of GFP

We first tested our modified pEAQ-HT constructs for GFP expression in *N. benthamiana* (Fig. 2). *N. benthamiana* is highly amenable to agroinfiltration and transient protein expression, and this is evident in our experiments with the pEAQ-HT constructs. Figure 2 shows high levels of GFP production in cytoplasm when agroinfiltrated with pEAQ-HT-DEST1-GFP-His (Fig. 2b), and in some cells, there is concentration of the green-fluorescent signal at the periphery of GFP-expressing cells despite not being actively targeted to the cell membrane or apoplast. The *Mt*PR1 secretion signal encoded in pEAQ-HT-DEST1-*Mt*PR1-GFP-His clearly leads to GFP export to the apoplast (Fig. 2c) with high levels of expression and concentration of green fluorescence signal at the margins of epidermal cells. Furthermore, granular and striated sub-cellular green fluorescence signals were observed and may represent newly synthesised GFP in the process of being targeted to the apoplast at various stages along the protein secretion pathway (Fig. 2c, inset image).

**Fig. 3:**
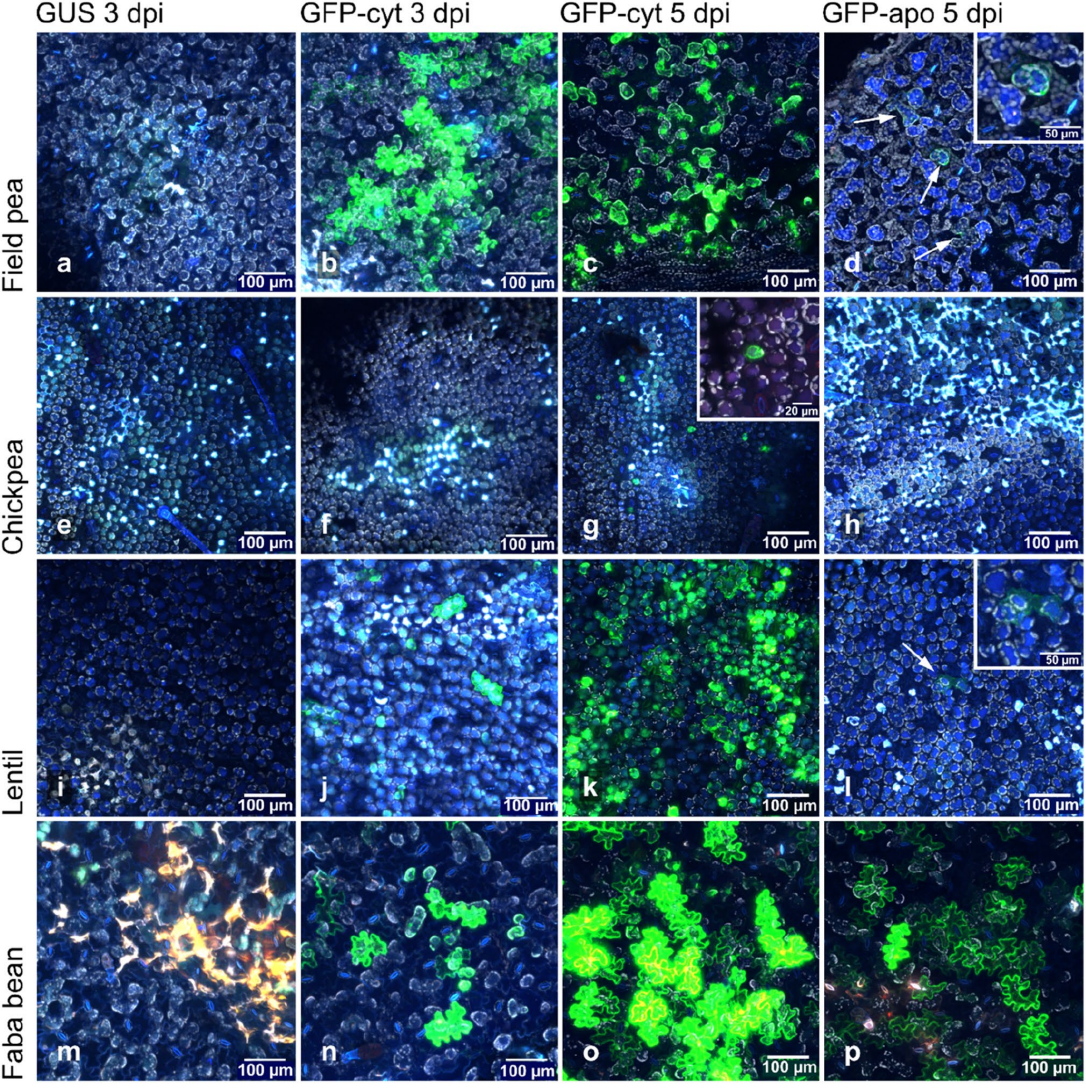
Agroinfiltration of grain legume species for the constitutive expression of GFP, observed using confocal microscopy. Leaves of Kaspa field pea (**a**–**d**), PBA HatTrick chickpea (**e**–**h**), PBA Ace lentil (**i**–**l**), and PBA Rana faba bean (**m**–**p**) were agroinfiltrated with *A. tumefaciens* strains carrying constructs for the expression of GUS control, cytoplasm-localised GFP (GFP_cyt_) or apoplast-targeted GFP (GFP_apo_). Confocal microscopy was performed at 3 and 5 days after infiltration and all images were collected using the 20 × objective. Green fluorescence of epidermal cells was clearly evident for field pea, lentil, and faba bean, but not for chickpea. GFP expression and export for faba bean (p) was observed when agroinfiltrated with the *A. tumefaciens* GFP_apo_ strains. Low-level GFP expression around occasional cells was observed for field pea (**d**) and lentil (**l**), as indicated with arrows and featured in inset images, but these observations were inconclusive as to the successful apoplast targeting of GFP in these species

Somatic cell transformation and transient expression in cool-season food legume species were tested by agroinfiltration of *A. tumefaciens* carrying the pEAQ-HT-DEST1-GFP-His and pEAQ-HT-DEST1-*Mt*PR1-GFP-His plasmid constructs, for validation of cytoplasm-localised and apoplast-targeted transient expression, respectively. Figure 3 shows confocal microscope imaging of field pea, chickpea, lentil, and faba bean (rows 1–4) agroinfiltrated for transient expression of GUS control (column 1), cytoplasm-localised GFP at 3 days post-infiltration (dpi) and 5 dpi (columns 2 and 3), and apoplast-targeted GFP (column 4). Levels of GFP expression at the site of infiltration and in the zone of perfusion of *Agrobacterium* suspension in field peas at 3 and 5 dpi were approximately the same. In some images, not all cells in the infiltration zone were transformed, particularly as observed at 3 dpi rather than for 5 dpi samples. It seems that there are varying rates at which cells can be transformed or variation in the efficiency of protein expression. In lentil and faba bean, the number of transformed cells was generally higher at 5 dpi than at 3 dpi, although the number of cells transformed was similar to that of field pea at 5 dpi (Fig. 3j, k and Fig. 3n, o). Images shown in Fig. 3 are representative of confocal observations of at least three infiltrated leaf sections. Indeed, levels of GFP transformation and expression in the large irregularly shaped epidermal cells in faba bean at 5 dpi were very efficient as shown by the intensity of GFP fluorescence in these samples (Fig. 3o). Transformation of chickpea leaves was observed at very low frequency with only occasional mesophyll cells showing evidence of GFP expression (Fig. 3g) at 5 dpi and no evidence of GFP expression at 3 dpi (Fig. 3f). We tested the ability of our modified pEAQ-HT-DEST1-*Mt*PR1-GFP-His construct to transform legume leaf cells (*Lc*PR1-GFP-His was used for lentil), and to express and traffic GFP to the apoplast (Fig. 3, column 4). At 5 dpi, green fluorescence around the margins of epidermal cells of agroinfiltrated faba bean leaves (Fig. 3p) indicated the likely effective export of protein to the apoplast and supported the likely utility of the pEAQ-HT-DEST1-*Mt*PR1-His vector for production and functional validation of apoplast-targeted candidate effector proteins in native host plants. In field pea (Fig. 3d) and lentil (Fig. 3l), there was weak evidence for low-level GFP expression and export, with green fluorescence at the margins of only occasional cells in leaves agroinfiltrated with constructs for apoplast-targeted GFP, carrying N-terminal *Mt*PR1 or *Lc*PR1 secretion signals.

**Fig. 4:**
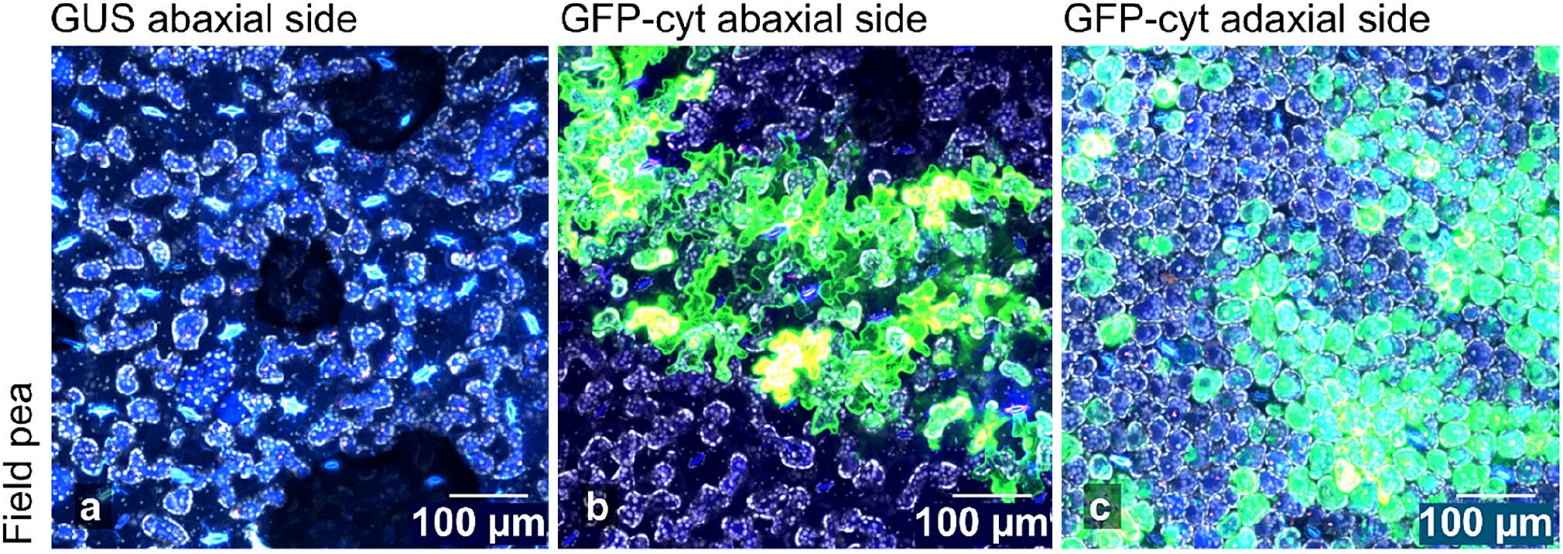
Agroinfiltration of field pea leaf tissue for the constitutive expression of (**a**) GUS control and (**b**, **c**) GFP at 5 days after infiltration, observed using confocal microscopy. Field pea leaves were infiltrated from the abaxial side and confocal imaging was performed both from the abaxial side into the tissue (**b**), and from the adaxial side (**c**). Imaging from the abaxial side towards the interior of the leaves shows that epidermal cells at the infiltration site were transformed and efficiently express GFP (**b**). Imaging from the adaxial side of leaves (**c**) shows that spherical and ovoid mesophyll cells of field pea are transformed and able to produce GFP

To characterise the success of agroinfiltration in legumes further, we assessed the transformation of cells across the leaf profile in field pea (Fig. 4). Our standard practice for agroinfiltration was to infiltrate *Agrobacterium* suspensions into the abaxial leaf surface where the stomata are more numerous. Confocal imaging from the abaxial side of the leaf (Fig. 4b) shows that the irregularly shaped epidermal cells close to the infiltration site are transformed efficiently. Imaging from the adaxial side shows that the agrobacteria are most likely distributed throughout the leaf profile beyond the epidermis and can readily transform cells in mesophyll layer, at the centre of the leaf (Fig. 4c). Although we did not attempt to monitor the distribution of agrobacteria, we infer the distribution of bacterial cells deep into the plant leaf tissue by the resulting transformation of cells within the different cell layers.

Field peas and faba beans were highly amenable to agroinfiltration and transient protein expression, while lentil and chickpea leaf cells were transformed at lower frequencies, particularly at 3 dpi (Fig. 3). Testing of apoplast-targeted NLP2 effector in legumes showed preliminary evidence to suggest lower efficacy of the agroinfiltration method for testing apoplast-targeted effector candidate proteins in lentil and chickpea. In our experience, the mechanical wounding of leaves from application of the plastic syringe and the perfusion of bacterial fluid at elevated pressure into leaf tissues is damaging to leaves. This damage causes non-specific chlorosis in some leaves after approximately 5 days, so it is important when testing effector candidate genes for their putative necrosis-inducing effects, to observe transgene-specific necrosis at around 3 dpi before background chlorosis develops. We tested several alternative lentil (Figure S1) and chickpea (Figure S2) varieties for expression of cytoplasm-localised GFP, and observed similar results to those for PBA Ace lentil (Fig. 3k) and PBA HatTrick chickpea (Fig. 3g). PBA Hurricane XT lentil transient expression of GFP was lower than that for PBA Ace and Nipper, which were similar to each other (Figure S1). Similar to the results shown in Fig. 3, the experiment in which different chickpea varieties were tested showed that PBA HatTrick performed the best out of the five varieties tested, Kyabra and PBA Seamer displayed evidence of infrequent mesophyll cell transformation, and Genesis 090 and PBA Monarch showed no evidence of transformation or transgene expression at five dpi.

### Comparison of GFP expression levels in *N. benthamiana* and field pea

**Fig. 5:**
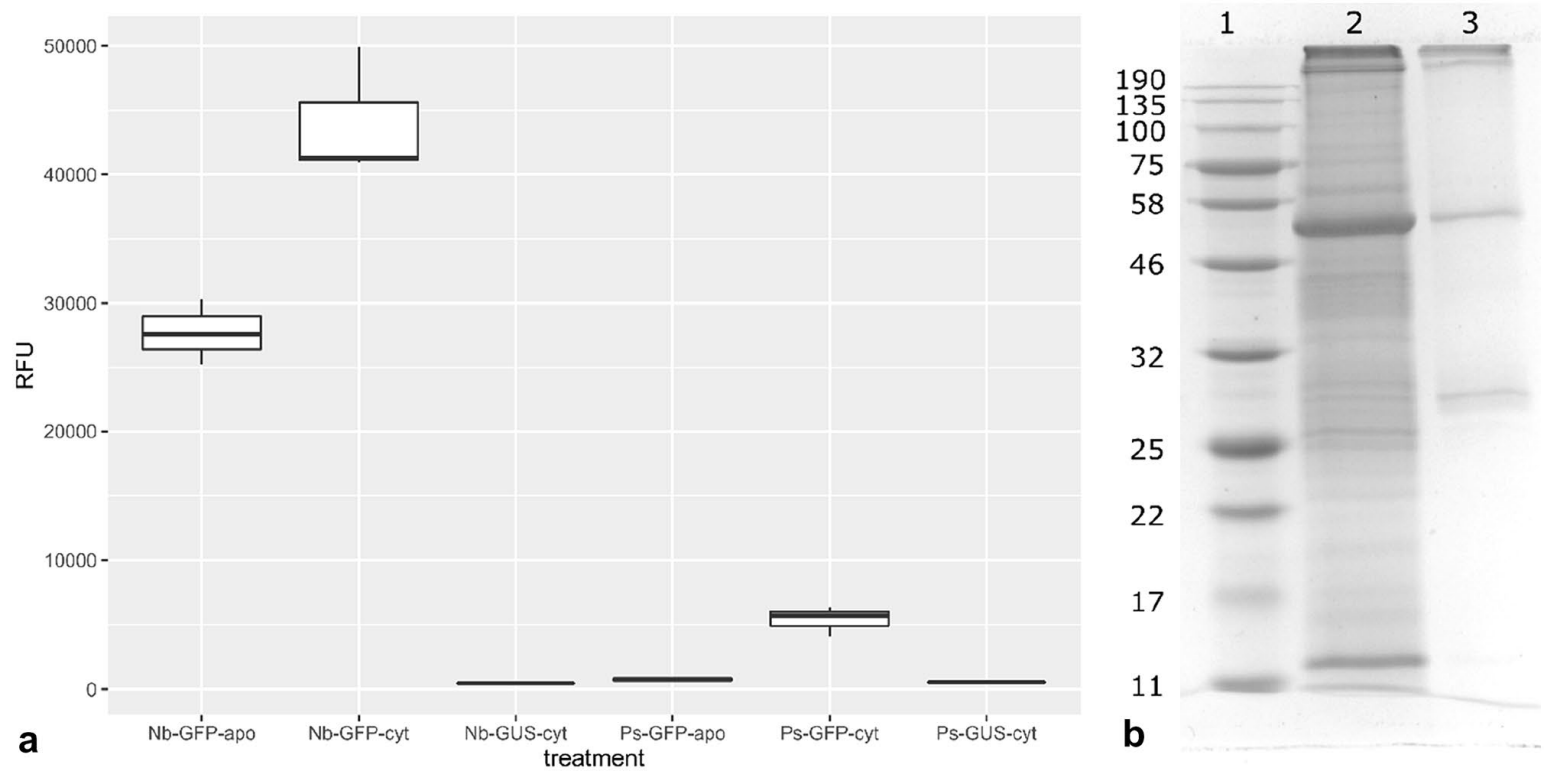
**a** Relative fluorescence units (RFU) of whole crude leaf extracts of 5-day agroinfiltrated leaf samples of *N. benthamiana* (Nb) and field pea (Ps) expressing recombinant GFP (apoplast-targeted—apo and cytoplasm-localised—cyt). Average fluorescence for cytoplasm-localised GFP in *N. benthamiana* was approximately tenfold higher than for field pea (*P* < 0.05). Apoplast targeted GFP fluorescence for *N. benthamiana* was 63% of the level for cytoplasm (*P* < 0.05). For field pea, cytoplasm-localised GFP fluorescence was significantly higher than the GUS control (*P* < 0.05) and the apoplast-targeted GFP fluorescence was 14% of the level for the cytoplasm-localised GFP sample. **b** SDS-PAGE of pooled extracts from nicotiana and pea for cytoplasm-localised GFP (lane 2) and nickel-NTA eluate containing partially purified GFP (27 KDa band; lane 3)

While the amount of GFP fluorescence could be semi-quantitatively measured using confocal microscopy and comparisons made visually by maintaining constant confocal settings, we made more accurate and quantitative assessment of the amount of GFP protein produced in nicotiana and field pea leaves by extracting the recombinant GFP from agroinfiltrated leaves after 5 days and measuring the amount of fluorescence for the two species for apoplast-targeted GFP and cytoplasm-localised GFP. We used cytoplasm-localised GUS expression as negative controls for the respective plant species. Figure 5 shows the relative fluorescence of plant extracts with the various transient expression treatments. Relative fluorescence values show that nicotiana produces around ten times the amount of GFP than field pea when agroinfiltrated with the expression construct for retention of expressed protein in the cytoplasm. When the protein is targeted to the apoplast, the relative fluorescence values are reduced. In nicotiana, relative fluorescence is reduced by approximately one-third. In field pea, targeting of the protein to the apoplast reduces the amount of fluorescence to around 14% of the values of the cytoplasm-localised GFP. To calibrate the amount of GFP protein per unit of measured relative fluorescence, we partially purified the recombinant protein using the 6x-histidine tag with affinity chromatography. SDS-PAGE shows the partially purified GFP with native molecular weight of 27 KDa (between the 25 and 32 KDa protein size standards). Band densitometry gave an estimate of 20% purity for the GFP band and Coomassie protein determination and correction for purity level gave an approximation of standard fluorescence intensity units for recombinant GFP of 11.4 RFU ng^−1^. GFP transient expression amounts on a per gram leaf tissue basis (wet weight) could then be calculated as 367 µg and 223 µg for cyt and apo expression, respectively, in *N. benthamiana*, and 46 µg and 2 µg for cyt and apo expression in field pea, respectively.

### Fungal effector NLP2 is targeted to the apoplast to induce necrosis in legume species

**Fig. 6:**
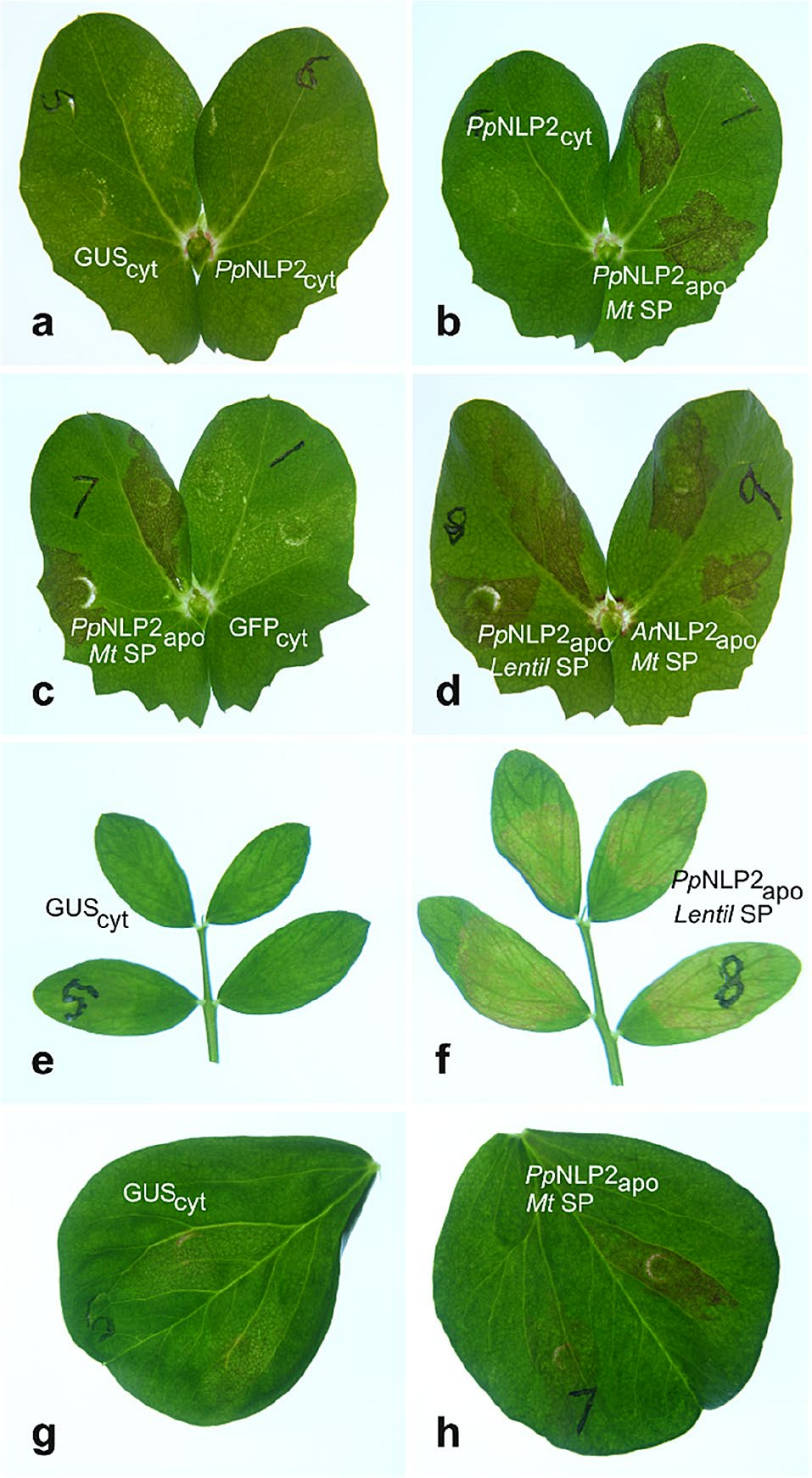
Agroinfiltration of grain legume species for the constitutive expression of necrosis and ethylene-inducing peptide (Nep1)-like protein (NLP2) from *P. pinodes* or *A. rabiei*, compared with GUS and GFP controls, at 5 days after infiltration. **a**–**d** Leaves of field pea, **e**, **f** lentil, and **g**, **h** faba bean were agroinfiltrated with *A. tumefaciens* strains carrying constructs for the expression of GUS and GFP controls, and cytoplasm or apoplast-localised NLP. Apoplast targeting of transiently expressed proteins was achieved by 5′-end fusion of signal peptide-encoding sequences of PR-1 genes from *M. truncatula* and *L. culinaris*, as indicated. Abbreviations: cyt, cytoplasm-localised; apo, apoplast-targeted; *Mt* SP, *Medicago* signal peptide; Lentil SP, lentil signal peptide; *Pp*, *P. pinodes*; *Ar*, *A. rabiei*

Cytoplasm-localised GFP has proved to be a reliable indicator of somatic cell transformation in field pea, lentil, and faba bean, with the number of cells and the level of transient protein expression clearly indicated by confocal fluorescence intensity. Apoplast targeting of GFP has been more difficult to assess with limited evidence of GFP export for field pea and lentil, but indication of efficient protein export for faba bean. A rigorous test of agroinfiltration for validating fungal pathogen effector candidates was to express the well-characterised necrosis-inducing effector NLP2 from legume pathogens, *P. pinodes* and *A. rabiei* (Fig. 6). Control GUS expression produced no necrosis in field pea (Fig. 6a), lentil (Fig. 6e) and faba bean (Fig. 6g). In field pea, cytoplasm-localised *P. pinodes* NLP2 (Fig. 6a, b) and GFP (Fig. 6c) similarly produced no necrosis reaction. In contrast, expression of *P. pinodes* and *A. rabiei* NLP2 localised to the apoplast by addition of either the *M. truncatula* (Fig. 6b, c) or lentil (Fig. 6d) PR-1 signal peptide routinely and reliably induced necrosis by 3 days after agroinfiltration; while expression of NLP2 with the *M. truncatula* signal peptide did not produce reliable necrosis for lentil with no observable necrosis compared with the GUS controls, using the PR-1 signal peptide sequence from lentil for lentil agroinfiltration produced a convincing although comparatively weak necrosis response (Fig. 6f). The necrosis response in faba bean for *P. pinodes* NLP2 targeted to the apoplast using the *M. truncatula* signal sequence was similar to the response in field pea using the same construct (Fig. 6h). The reported evidence of NLP only generating necrosis when the protein is delivered to the apoplast side of the plasma membrane in dicot plants (Qutob et al. 2006) is essential to our contention that the clear necrosis in field pea and faba bean leaves when using pEAQ constructs with signal peptide-encoding sequence is indicative of successful delivery of protein to the apoplast when using these constructs.

## Discussion

Characterisation of the roles of fungal phytopathogen effectors in plant disease has often employed methods in which recombinant proteins produced in *E. coli* or yeast are infiltrated into host leaf tissue to assess necrosis or hypersensitive responses. For example, effectors from the wheat pathogen *Parastagonospora nodorum* have been produced in *E. coli* (Zhang et al. 2017) and *Pichia pastoris* (Liu et al. 2009, 2012) expression systems at concentration and purity levels high enough for subsequent functional and structural analysis. Effector protein studies in which recombinant proteins are infiltrated into host tissues are often efficient and successful. However, correct protein folding, formation of disulphide linkages, and potentially post-translational modifications are essential for the proper biological function of *bona fide* effectors in their corresponding sensitive plant hosts, and these factors are not always assured for recombinant microbial proteins. It can never be certain whether a lack of hypersensitivity or necrosis response to an infiltrated effector candidate is due to incorrect protein conformation, insensitivity of the host species, or cultivar to a particular effector, or that the candidate protein is not a genuine effector. Transient expression of effectors using the agroinfiltration method is an alternative strategy for validating candidate effectors from plant pathogens and for functional studies of confirmed effectors. The co-option of the host plant protein synthesis machinery in transient expression gives better assurance of native function of the expressed protein than for recombinant bacterial or yeast proteins. Furthermore, effector candidate synthesis within the plant to be tested for sensitivity eliminates the need for protein extraction and purification, additional steps that can result in loss of biological activity.

Agroinfiltration in *Nicotiana* spp. has been used to investigate effectors from broad host range pathogens, such as *Phytophthora capsici*, in which necrosis and hypersensitivity in the test plant indicates a role in disease (Mafurah et al. 2015; Chen et al. 2018). Studies of host-specific avirulence effectors in *N. benthamiana* require co-expression of the cognate receptor from the native host of the pathogen from which the effector originates. Examples include studies of *Verticillium* and *Cladosporium* effectors and their corresponding receptors in tomato (Van der Hoorn et al. 2000; Zhang et al. 2013, 2014). These types of receptor-effector interaction studies use *Nicotiana* spp*.* as a platform for investigating pathogen virulence and associated host defence responses in an exogenous system. For the study of fungal virulence genes of host-specific pathogens such as *Ascochyta* spp., in the absence of knowledge about resistance or susceptibility receptors, we need robust methods for transient expression of effector candidates in the native host of the pathogen in question. Agroinfiltration has been demonstrated for legume species, including common bean (*Phaseolus vulgaris*; Kapila et al., 1997; Suzaki et al., 2019), soybean, and pea (*Pisum sativum*; Guy et al., 2016; Klosterman et al., 2003; Van der Hoorn et al., 2000). Our study presents additional data for field pea that corroborates these earlier reports, and tests the method for faba bean, lentil, and chickpea.

With the growing availability of whole genome sequences of plant pathogen species and the development of protein effector candidate prediction programs (Sperschneider et al. 2015, 2016, 2018; Jones et al. 2018), molecular plant pathologists require efficient transient expression methods for testing and validating candidate genes for their role in plant diseases. Although recent advances have been made in wheat transformation (Hayta et al. 2019), wheat and likely other cereal crop species hosts are less amenable to genetic transformation required for *Agrobacterium*-mediated transient expression. Therefore, in vitro expressions in *E. coli* and yeast are suitable for effector studies in wheat and barley pathosystems. In contrast, dicot species are generally routinely genetically transformed (Singh and Prasad 2016). Thus, transient expression by agroinfiltration is a practical effector candidate validation strategy for fungal pathogens of dicot plants, such as in our case, for ascochyta blights of cool-season grain legumes.

Transient expression of GFP in this study has proven to be a reliable marker of both the efficiency of transformation of plant cells of the host species tested and the efficiency of transgene expression. Confocal microscopy with consistent excitation laser and PMT detection settings has enabled precise localisation of transformed cells and semi-quantitative evaluation of protein synthesis in the different cell types and species used in the research. Imaging from abaxial and adaxial sides of leaves shows that both the epidermal and mesophyll cells are transformed and efficiently produce the transgene-encoded protein. This demonstrates that sufficient amounts of effector candidate proteins can be produced and distributed across the leaf profile to generate the necrosis response that characterises the disease state in a natural infection. The overall findings in the study were that the somatic cells in the zone of agroinfiltration, of field pea, lentil, and faba bean leaves, can be genetically transformed and transgenes under the control of the CaMV-35S promoter are readily expressed within 3–5 days after agroinfiltration. The levels of gene expression in pea, lentil, and faba bean were lower than in *N. benthamiana*, widely considered to be the most suitable plant species for transformation and transient expression (Bally et al. 2018). Protein extraction, partial purification from agroinfiltrated leaves, and subsequent quantitation using GFP fluorescence enable a reasonable approximation of the relative transformation and expression efficiencies of field pea and nicotiana, and these corroborate the visual assessments by confocal microscopy. In addition, we have shown that incorporation of transgene sequence that encodes the signal peptide for the secreted disease response protein, PR-1 from *M. truncatula*, leads to targeting of expressed protein to the apoplast. This observation was most evident for *N. benthamiana* in which GFP was concentrated at epidermal cell margins as an indication of successful export of protein from transformed cells.

Apoplast targeting was also evident for faba bean, but for field pea and lentil, observation of GFP at the margins of epidermal and mesophyll cells was not conclusive. Quantitation of GFP from agroinfiltrated leaf extracts showed that there was less GFP recovered from the field pea samples expressing apoplast-targeted GFP than the cytoplasm-localised GFP-infiltrated field pea leaves. This is perhaps due to rapid degradation of the protein in the apoplast and constant protein turnover in contrast to the possible accumulation of the protein when retained in the cytoplasm. For faba bean and field pea, the efficient expression and export of the *P. pinodes* NLP2 effector to the apoplast were assumed by abundant leaf necrosis, evident at 3 days after infiltration. In spite of the possible degradation of apoplast-delivered NLP2 in field pea, the protein appears to remain active long enough to cause the expected necrosis of host tissue. Apoplast targeting of neither GFP nor the secondary necrosis effects of protein export for NLP2 fused to an N-terminal *M. truncatula* signal peptide were observed in lentil, despite the likely transformation and transgene expression as shown for cytoplasm-localised GFP. NLP effectors almost invariably carry a secretion signal peptide and are secreted by invading pathogens to the apoplast of susceptible host plants where the effector is perceived by cell surface receptors exposed to the apoplast (Gijzen and Nurnberger 2006; Qutob et al. 2006). With the possibility that the medicago signal sequence function was defective in lentil, a construct for lentil transient expression with an endogenous lentil PR-1 signal peptide was tested. Although confocal microscopy evidence for GFP targeting in lentil was questionable, expressed *P. pinodes* NLP2 targeted to the lentil apoplast by the lentil PR-1 signal sequence produced observable necrosis and we determined this to be sufficient evidence of the anticipated effector function in lentil. The lentil PR-1 sequence was found by a tBLASTn search of the Cool Season Food Legume, *Lens culinaris* RefTrans v1 database (https://www.pulsedb.org) with the medicago sequence as query. The amino acid sequences of the *M. truncatula* and lentil PR-1 proteins are 41% identical. A signal peptide sequence was predicted for both proteins using SignalP 5.0, although there were only three amino acid identities in the predicted signal peptide sequences of 23 and 21 amino acids for medicago and lentil PR-1 sequences, respectively. It seems that these differences most likely account for the unsuccessful *Mt*PR1 pEAQ constructs for apoplast targeting in lentil and that future effector candidate testing and validation studies in lentil should use the pEAQ construct with the native lentil signal sequence. The pathogenesis-related PR-1 proteins from plants are some of the most highly expressed proteins in response to plant pathogen infection and are targeted to the apoplast where they presumably exert their role in plant defence against microbial pathogens (Breen et al. 2017). PR-1 was chosen as the model sequence for a canonical signal peptide sequence and *M. truncatula* was seen as close enough to provide a signal sequence that would function across the legume species in the study. This was not to be the case for lentil, however, and the alternative lentil sequence was found to be effective in our agroinfiltration construct.

Chickpea proved to be unsuitable for agroinfiltration with very low transformation efficiencies or perhaps inefficient transgene expression under the CaMV-35S promoter used in the study. Recent research on transformation and transgene expression efficiency in *N. benthamiana* for transgenes controlled by the CaMV-35S promoter suggests that DNA methylation of the transgene promoter may lead to low transcription efficiency (Philips et al. 2019). Testing of multiple cultivars of lentil and chickpea showed that lentil is likely widely amenable to the agroinfiltration method for transient expression and that chickpea is likely to be unsuitable, with none of the cultivars tested being better than the poorly performing PBA HatTrick, and PBA Monarch and Genesis 090 showing no evidence of GFP expression. Our results show that GFP fluorescence in occasional chickpea cells, particularly in the variety PBA HatTrick, was comparable to the more successful legume species such as field pea and lentil. It is unclear whether our constructs failed to produce uniform genetic transformation of leaf cells in the zone of infiltration or whether transcriptional inactivation is the reason for the failure of agroinfiltration in chickpea. Future studies for chickpea will aim to improve the agroinfiltration process with testing of native constitutive promoters rather than the usual CaMV-35S promoter.

Agrobacterium can express protein under control of the CaMV-35S promoter (Vancanneyt et al. 1990) under some circumstances, and our study has not attempted to determine whether the GFP and NLP2 proteins are produced exclusively by the transformed host plant cells or whether there is the possibility that some protein could be from the bacterium. One could contend that agrobacterium infiltrated to the plant apoplast could synthesise the target proteins and where there is a plant secretion signal peptide encoded in the transgene, the protein could be exported to the cytoplasm. From the clear cytoplasm localisation of GFP where no secretion signal was included in the expression construct, we conclude that plant cells are the most abundant source of the transgene-encoded protein. We do not rule out the possibility that a proportion of the protein could be produced by infiltrated *A. tumefaciens*; however, the origin of the protein is not essential to the method. It is the localisation of the recombinant fungal effector candidate protein in the apoplast that is key to this experimental method and its use in testing necrosis-causing capability and, thus, inferring a role for fungal proteins in causing plant disease.

In the current work, we have demonstrated the suitability of the agroinfiltration method for the high-throughput functional screening of *Ascochyta* spp. effector candidates in grain legumes, with novel findings for faba bean and lentil, and for chickpea, we conclude that further construct development is required. The Gateway-compatible pEAQ-HT-DEST1 transient expression vector developed by Sainsbury and Lomonossoff (Sainsbury et al. 2009; Peyret and Lomonossoff 2013) has provided a robust vector system that ensures high levels of protein expression in plants. Our addition of specific secretion signal-encoding sequences has produced an efficient system for fungal effector studies field pea, lentil, and faba bean. With further construct development, similar agroinfiltration methods for chickpea will become available and will enable efficient and reliable testing of effector candidate genes from *Ascochyta* and other disease-causing fungi of grain legumes.

## Supplementary Information

Below is the link to the electronic supplementary material.

Supplementary file1 (DOCX 14 KB)

Supplementary file2 (DOCX 5302 KB)

Supplementary file3 (DOCX 16 KB)
