# Supplementary material for: Agroinfiltration for transient gene expression and characterisation of fungal pathogen effectors in cool-season grain legume hosts
Source: Plant Cell Rep. 2021 Apr 3;40(5):805–18. doi: 10.1007/s00299-021-02671-y (PMC8058004; doi:10.1007/s00299-021-02671-y)
Supplement: Supplementary file 1 — Supplementary file1 (DOCX 14 KB) [file 299_2021_2671_MOESM1_ESM.docx]

**Supplementary Table Table_S1**

| **Description** | **Name** | **Sequence** |
| --- | --- | --- |
| Step 1 and 3 His tag PCR fwd | JD013 | TAGGCGCGCCAAGCTTGAGACTCTA |
| Step 2 His tag PCR fwd | JD014 | ACTAGTGATGGTGATGGTGATGAACCACTTTGT |
| Step 2 and 3 His tag PCR rev | JD015 | TAGGTCGACCATAGTGACTGGATA |
| Step 1 His tag PCR rev | JD016 | ATCACCATCACCATCACTAGTCCTTTAACTCT |
| Universal attB1 | JD017 | GGGGACAAGTTTGTACAAAAAAGCAGGCT |
| Universal attB2 | JD018 | GGGGACCACTTTGTACAAGAAAGCTGGGT |
| GFP specific fwd | attB1 GFP | AAAAAGCAGGCTCCATGGTGAGCAAGGGCGAG |
| GFP specific rev | attB2 GFP | AGAAAGCTGGGTACTTGTACAGCTCGTCCATG |
| *P. pinodes* NLP1 fwd | JD023 | AAAAAGCAGGCTCCATGGTGCCAACAAGCTTG |
| *P. pinodes* NLP1 rev | JD024 | AGAAAGCTGGGTGGAAGTAAGCATCCGCAAGG |
| *P. pinodes* NLP2 fwd | JD025 | AAAAAGCAGGCTCCATGTCACCTACTCCTGCC |
| *P. pinodes* NLP2 rev | JD026 | AGAAAGCTGGGTGGACCCAAGCCTTGTCGAGG |
| *A. rabiei* NLP2 fwd | JD239 | AAAAAGCAGGCTCCATGTCTCCCACACCATCT |
| *A. rabiei* NLP2 rev | JD240 | AGAAAGCTGGGTAGACGAAAGCGTTGTC |
| Colony PCR pEAQ vector fwd | JD029 | AAAACCGCTCACCAAACATA |
| Colony PCR pEAQ vector rev | JD030 | TTTTCTTTGAAACAGAGTTTTCC |

Table S1 Primers used for the production of modified pEAQ agroinfiltrarion constructs, including: Three-step His-tag fragment PCR; Universal attB1 and attB2 primers for PCR amplification of DNA from gene specific PCR products and addition of attB1 and attB2 Gateway recombination sites; gene specific primers for amplification of coding sequences for GFP and fungal NLP effectors, and containing the attB1 and attB2 primer annealing sites for the universal primers; pEAQ vector primers for colony PCR detection of successful *A. tumefaciens* transformants, with primer binding to transgene sequence outside the attB1 and attB2 sites, and within the construct sequence between right and left border sequences. Complementary nucleotide sequence for primer binding in the two-step PCR reactions are underlined.
