# Supplementary material for: Agroinfiltration for transient gene expression and characterisation of fungal pathogen effectors in cool-season grain legume hosts
Source: Plant Cell Rep. 2021 Apr 3;40(5):805–18. doi: 10.1007/s00299-021-02671-y (PMC8058004; doi:10.1007/s00299-021-02671-y)
Supplement: Supplementary file 2 — Supplementary file2 (DOCX 5302 KB) [file 299_2021_2671_MOESM2_ESM.docx]

**
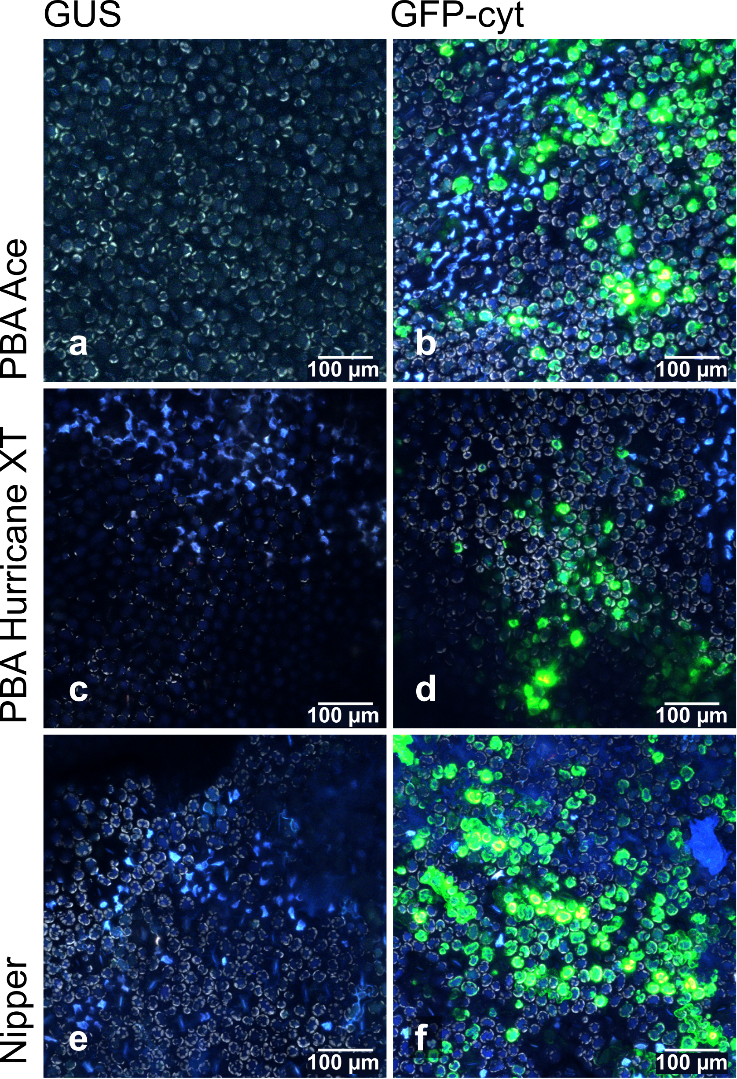
**

Figure S1 Agroinfiltration of lentil varieties for the constitutive expression of GUS control or cytoplasm-localised GFP (GFP-cyt), observed using confocal microscopy at five days after infiltration.

**
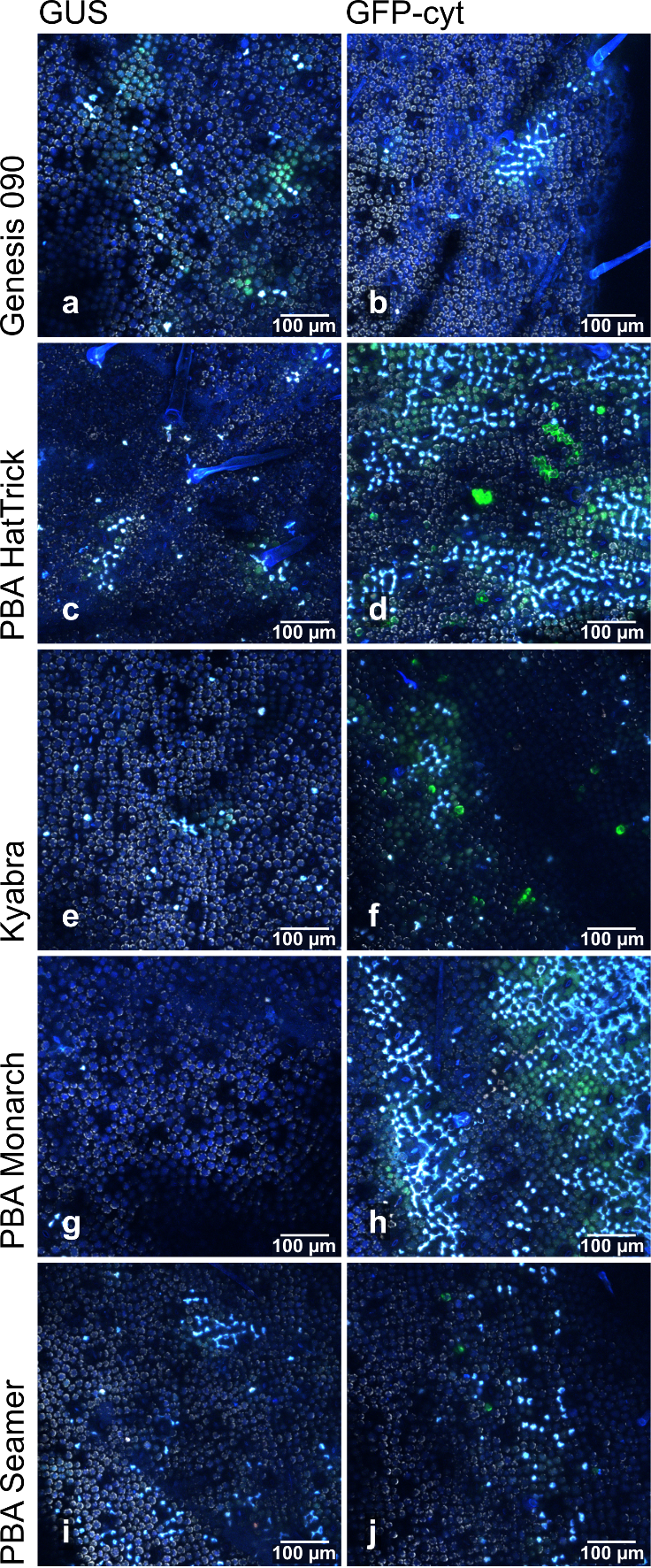
**

Figure S2 Agroinfiltration of chickpea varieties for the constitutive expression of GUS control or cytoplasm-localised GFP (GFP-cyt), observed using confocal microscopy at five days after infiltration.
