# Supplementary material for: Agroinfiltration for transient gene expression and characterisation of fungal pathogen effectors in cool-season grain legume hosts
Source: Plant Cell Rep. 2021 Apr 3;40(5):805–18. doi: 10.1007/s00299-021-02671-y (PMC8058004; doi:10.1007/s00299-021-02671-y)
Supplement: Supplementary file 3 — Supplementary file3 (DOCX 16 KB) [file 299_2021_2671_MOESM3_ESM.docx]

**Supplementary File File_S1:**

Insert sequences of cDNAs used for the construction of modified pEAQ-HT-DEST1 constructs for expression of GFP and NLP2 effectors from *Peyronellaea pinodes* and *Ascochyta rabiei*. Underlining of DNA sequence indicates the location of primer sites and attB1 and attB2 sites for directional Gateway cloning to the Gateway entry vector and subsequent transfer to modified pEAQ vectors for transient expression in plants.

>GFP_flanked_by_attB1_and_attB2_sites

GGGGACAAGTTTGTACAAAAAAGCAGGCTCCATGGTGAGCAAGGGCGAGGAGCTGTTCACCGGGGTGGTGCCCATCCTGGTCGAGCTGGACGGCGACGTAAACGGCCACAAGTTCAGCGTGTCCGGCGAGGGCGAGGGCGATGCCACCTACGGCAAGCTGACCCTGAAGTTCATCTGCACCACCGGCAAGCTGCCCGTGCCCTGGCCCACCCTCGTGACCACCCTGACCTACGGCGTGCAGTGCTTCAGCCGCTACCCCGACCACATGAAGCAGCACGACTTCTTCAAGTCCGCCATGCCCGAAGGCTACGTCCAGGAGCGCACCATCTTCTTCAAGGACGACGGCAACTACAAGACCCGCGCCGAGGTGAAGTTCGAGGGCGACACCCTGGTGAACCGCATCGAGCTGAAGGGCATCGACTTCAAGGAGGACGGCAACATCCTGGGGCACAAGCTGGAGTACAACTACAACAGCCACAACGTCTATATCATGGCCGACAAGCAGAAGAACGGCATCAAGGTGAACTTCAAGATCCGCCACAACATCGAGGACGGCAGCGTGCAGCTCGCCGACCACTACCAGCAGAACACCCCCATCGGCGACGGCCCCGTGCTGCTGCCCGACAACCACTACCTGAGCACCCAGTCCGCCCTGAGCAAAGACCCCAACGAGAAGCGCGATCACATGGTCCTGCTGGAGTTCGTGACCGCCGCCGGGATCACTCTCGGCATGGACGAGCTGTACAAGTACCCAGCTTTCTTGTACAAAGTGGTCCCC

>P_pinodes_NLP2_flanked_by_attB1_and_attB2_sites

GGGGACAAGTTTGTACAAAAAAGCAGGCTCCATGTCACCTACTCCTGCCACGCTTGATGCTCGTGGTACGGTCCCCCACGACTCCTTGAACCCTCTTCCGAACCGCCTGCAAAATGGAGTTGTCGGTCGTGCAATCGAGAAGTTCACTCCTCTCCTTCATATTGCTCATGGCTGCCAGCCATACACCGCAGTTGACAATGCGGGTAACACCAGTGGAGGGCTTCAAGACACCGGCAACCCCAGCGCTGGCTGCGGTGACGGCAGGAAGGGCCAAATCTACGCTCGTGGCGCCTGGCACAAGGGCAGGTTCGCCATCATGTATGCCTGGTACTTTCCCAAGGACCAGCCGATCGCCGGCAACGTTGCAGGCGGCCACCGTCACGACTGGGAGAGCGTCGTCGTCTGGATTGACAACCCCGCCAACGCCAACCCTCGCATTCTGGGAGCTGCTGCATCGGGCCATGGCGGGTACAAGAAGTCCACCAACCCCCAGCGTCAGGGCAACAACGTGAAGGTCGAATACTTCACTAGATTCCCTACCAACCATGAGCTTCAGTTCACGAACACTGTTGGCAGGTCGTACTGGATCCAGGACTGGAACGCTATGCCTGCCAATGTCCGCAACGTCCTTTCCCAACCCAACCTCTTTGGAAAAGGGAACGTTCCTTTCAGGCCCGACAACTTCAACGGCAACCTCGACAAGGCTTGGGTCCACCCAGCTTTCTTGTACAAAGTGGTCCCC

>A_rabiei_NLP2_flanked_by_attB1_and_attB2_sites

GGGGACAAGTTTGTACAAAAAAGCAGGCTCCATGTCTCCCACACCATCTGAACTCGATGCTCGCGCTGTAGTCAACCATGACTCTTTGAATCCGATCCATACCCGTGTGCAAGGTGGAGCGATTGGACGTGCTATCGAGAGGTTCCAACCCTTACTCCACATCGCCCACGGTTGTCAGCCATACACTGCTGTTGATGATGCCGGAAATACCAGTGGTGGTCTCCAGGATACTGGCAACGTCAGTGCTGGGTGCCGAGACCCCAACAAGGGTCAGACCTACGTCAGAGCGGCCTGGCACAAGGGCAAGTTCGCCATGATGTTCGCTTGGTACTGGCCGAAGGATCAACCAGCCGCTGGCAACGTTGCTGGCGGTCACCGTCACGATTGGGAAAACGTTGTCGTTTGGATAGACAACCCCTCGAACGCCAATCCCCGTATTCTAGGCGCCGCTGCATCTGCCCATGGTGGTTACGCGCCTACTTCTACCCCAAACAGGAGAGGCGACAATGTCCTAGTAGAATACTTTGTCGAATTCCCAAGGAACCATGCGCTTCAATTCACTGAAACAGTTGGACGTACTTACTGGATCAGCGATTACGATGTTATGCCCAACGCGGAGAAACAAGCTCTCGCAAGCACAACAATCTTCGGAGACGCAAACGTGCCTTTCAGGCCTGATAACTTCGCCACCAACTTGGACAACGCTTTCGTCTACCCAGCTTTCTTGTACAAAGTGGTCCCC
